# Supplementary material for: Health-Related Quality of Life and Side Effects in Gastrointestinal Stromal Tumor (GIST) Patients Treated with Tyrosine Kinase Inhibitors: A Systematic Review of the Literature
Source: Cancers (Basel). 2022 Apr 5;14(7):1832. doi: 10.3390/cancers14071832 (PMC8997462; doi:10.3390/cancers14071832)
Supplement: Supplementary file 1 [file cancers-14-01832-s001.zip › cancers-1639211-supplementary.pdf]

Supplementary Materials

Supplementary File S1. Search String

1. GIST

1.1. Medline

Gastrointestinal Stromal Tumors/ or (gastrointestinal stromal neoplasm* OR gastrointestinal stromal sarcoma* OR gastrointestinal stromal tumo* OR gastro-intestinal stromal neoplasm* OR gastro-intestinal stromal sarcoma* OR gastro-intestinal stromal tumo* OR GIST OR GISTs).ti,ab,kf.

1.2. Embase

Gastrointestinal Stromal Tumors/ or (gastrointestinal stromal neoplasm or gastrointestinal stromal sarcoma* or gastrointestinal stromal tumo* or gastro-intestinal stromal neoplasm* or gastro-intestinal stromal sarcoma* or gastro-intestinal stromal tumo* or GIST or GISTs).ti,ab,kw.

1.3. PsycINFO

(gastrointestinal stromal neoplasm* or gastrointestinal stromal sarcoma* or gastrointestinal stromal tumo* or gastro-intestinal stromal neoplasm* or gastro-intestinal stromal sarcoma* or gastro-intestinal stromal tumo* or GIST or GISTs).tw.

1.4. Web of Science

("gastrointestinal stromal neoplasm*" or "gastrointestinal stromal sarcoma*" or "gastrointestinal stromal tumo*" or "gastro-intestinal stromal neoplasm*" or "gastro-intestinal stromal sarcoma*" or "gastro-intestinal stromal tumo*" or GIST or GISTs)

1.5. Cochrane

(gastrointestinal stromal neoplasm* or gastrointestinal stromal sarcoma* or gastrointestinal stromal tumo* or gastro-intestinal stromal neoplasm* or gastro-intestinal stromal sarcoma* or gastro-intestinal stromal tumo* or GIST or GISTs):ti,ab,kw

2. TKI

2.1. Medline

exp Protein Kinase Inhibitors/ or (imatinib OR glivec OR gleevec OR sunitinib OR sutent OR ST 1571 OR ST1571 OR STI571 OR STI-571 OR CPG57148 OR CPG-57148 OR CPG57148B OR SU11248 OR SU-11248 OR SU011248 OR SU-0111248 OR regorafenib OR Stivarga OR avapritinib OR AYVAKIT OR BLU-285 OR BLU285 OR ripretinib OR DCC-2618 OR DCC2618 OR Qinlock OR entrectinib OR Rozlytrek OR protein kinase inhibitor* OR TKI* OR tyrosine kinase inhibitor*).ti,ab,kf.

2.2. Embase

exp protein tyrosine kinase inhibitor/ or (imatinib OR glivec OR gleevec OR sunitinib OR sutent OR ST 1571 OR ST1571 OR STI571 OR STI-571 OR CPG57148 OR CPG-57148 OR CPG57148B OR SU11248 OR SU-11248 OR SU011248 OR SU-0111248 OR regorafenib OR Stivarga OR avapritinib OR AYVAKIT OR BLU-285 OR BLU285 OR ripretinib OR DCC-2618 OR DCC2618 OR Qinlock OR entrectinib OR Rozlytrek OR protein kinase inhibitor* OR TKI* OR tyrosine kinase inhibitor*).ti,ab,kw,tn.

2.3. PsycINFO

(imatinib OR glivec OR gleevec OR sunitinib OR sutent OR ST 1571 OR ST1571 OR STI571 OR STI-571 OR CPG57148 OR CPG-57148 OR CPG57148B OR SU11248 OR SU-11248 OR SU011248 OR SU-0111248 OR regorafenib OR Stivarga OR avapritinib OR

AYVAKIT OR BLU-285 OR BLU285 OR ripretinib OR DCC-2618 OR DCC2618 OR Qinlock OR entrectinib OR Rozlytrek OR protein kinase inhibitor* OR TKI* OR tyrosine kinase inhibitor*).tw.

2.4. Web of Science

(imatinib OR glivec OR gleevec OR sunitinib OR sutent OR "ST 1571" OR ST1571 OR STI571 OR STI-571 OR CPG57148 OR CPG-57148 OR CPG57148B OR SU11248 OR SU-11248 OR SU011248 OR SU-0111248 OR regorafenib OR Stivarga OR avapritinib OR AYVAKIT OR BLU-285 OR BLU285 OR ripretinib OR DCC-2618 OR DCC2618 OR Qinlock OR entrectinib OR Rozlytrek OR "protein kinase inhibitor*" OR TKI* OR "tyrosine kinase inhibitor*")

2.5. Cochrane

(imatinib OR glivec OR gleevec OR sunitinib OR sutent OR ST 1571 OR ST1571 OR STI571 OR STI-571 OR CPG57148 OR CPG-57148 OR CPG57148B OR SU11248 OR SU-11248 OR SU011248 OR SU-0111248 OR regorafenib OR Stivarga OR avapritinib OR AYVAKIT OR BLU-285 OR BLU285 OR ripretinib OR DCC-2618 OR DCC2618 OR Qinlock OR entrectinib OR Rozlytrek OR protein kinase inhibitor* OR TKI* OR tyrosine kinase inhibitor*):ti,ab,kw

3. QoL

3.1. Medline

exp "Activities of Daily Living"/ or exp Anxiety/ or Depression/ or exp Disability Evaluation/ or exp Fatigue/ or exp fear/ or exp Health Status/ or exp Pain/ or physical fitness/ or Psychological Distress/ or exp "Surveys and Questionnaires"/ or Quality-Adjusted Life Years/ or "Quality of Life"/ or "International Classification of Functioning, Disability and Health"/ or "Value of Life"/ or exp qualitative research/ or exp Focus Groups/ or Interviews as Topic/

((activit* ADJ2 daily living) or ADL or adjusted life year* or anxiety or (limit* ADJ2 activit*) or daily activit* or daily functioning or daily life activit* or daly* or depress* or disability adjusted life or disability assessment* or disability evaluation* or distress* or disease assessment* or disease burden or disease evaluation* or emotional or fatigue or fear or functional status or health assessment* or health evaluation* or (health adj3 (utilit* or status)) or health perception* or health outcome* or hql or hqol or health risk or hrqol or HRQL or healthy years equivalent* or independent living or International Classification of Functioning or life satisfaction or mental health or panic or patient reported or patient based or performance status or pain or psychosocial or psychological or physical fitness or physical function* or Qaly* or Qol or (qualit* adj3 life) or self-care or self-neglect or social participation or self-reported or sexual or social functioning or symptom burden or symptom assessment* or vitality or well-being or wellbeing or qualitative research or qualitative stud* or focus group* or interview* or survey* or questionnaire*).ti,ab,kf.

3.2. Embase

ADL disability/ or daily life activity/ or exp disease burden/ or exp depression/ or exp fatigue/ or exp fear/ or exp health status/ or exp health survey/ or exp general health status assessment/ or exp general mental disease assessment/ or exp pain/ or exp "quality of life"/ or exp qualitative research/ or exp questionnaire/ or exp interview/ or ((activit* adj2 daily living) or ADL or adjusted life year* or anxiety or (limit* ADJ2 activit*) or daily activit* or daily functioning or daily life activit* or daly* or depress* or disability adjusted life or disability assessment* or disability evaluation* or distress* or disease assessment* or disease burden or disease evaluation* or emotional or fatigue or fear or functional status or health assessment* or health evaluation* or (health adj3 (utilit* or status)) or health perception* or health outcome* or hql or hqol or health risk or hrqol or HRQL or healthy years equivalent* or independent living or International Classification of

Functioning or life satisfaction or mental health or panic or patient reported or patient based or performance status or pain or psychosocial or psychological or physical fitness or physical function* or Qaly* or Qol or (qualit* adj3 life) or self-care or self-neglect or social participation or self-reported or sexual or social functioning or symptom burden or symptom assessment* or vitality or well-being or wellbeing or qualitative research or qualitative stud* or focus group* or interview* or survey* or questionnaire*).ti,ab,kw.

3.3. PsycINFO

-

3.4. Web of Science

((activit* NEAR/1 daily living) or ADL or "adjusted life year*" or anxiety or (limit* NEAR/1 activit*) or "daily activit*" or "daily functioning" or "daily life activit*" or daly* or depress* or "disability adjusted life" or "disability assessment*" OR "disability evaluation*" or distress* or "disease assessment*" or "disease burden" or "disease evaluation*" or emotional or fatigue or fear or "functional status" or "health assessment*" or "health evaluation*" or (health NEAR/2 (status OR utilit*)) or "health perception*" or "health outcome*" or hql or hqol or "health risk" or hrqol or HRQL or "healthy years equivalent*" or "independent living" or "International Classification of Functioning" or "life satisfaction" or "mental health" or panic or "patient reported" or "patient based" or "performance status" or pain or psychosocial or psychological or "physical fitness" or "physical function*" or Qaly* or Qol or (qualit* NEAR/2 life) or self-care or self-neglect or "social participation" or self-reported or sexual or "social functioning" or "symptom burden" or "symptom assessment*" or vitality or well-being or wellbeing or "qualitative research" or "qualitative stud*" or "focus group*" or interview* or survey* or questionnaire*)

3.5. Cochrane

(ADL or adjusted life year* or anxiety or limit* activit* or daily activit* or daily functioning or daly* or depress* or disability adjusted life or disability assessment* or disability evaluation* or distress* or disease assessment* or disease burden or disease evaluation* or emotional or fatigue or fear or functional status or health assessment* or health evaluation* or health utilit* or health status or health perception* or health outcome* or hql or hqol or health risk or hrqol or HRQL or healthy years equivalent* or independent living or International Classification of Functioning or life satisfaction or mental health or panic or patient reported or patient based or performance status or pain or psychosocial or psychological or physical fitness or physical function* or Qaly* or Qol or life quality or self-care or self-neglect or social participation or self-reported or sexual or social functioning or symptom burden or symptom assessment* or vitality or well-being or wellbeing or
qualitative research or qualitative stud* or focus group* or interview* or survey* or questionnaire*):ti,ab,kw

4. Side effects

4.1. Medline

exp "Drug-Related Side Effects and Adverse Reactions"/ OR Long Term Adverse Effects/ OR Adverse Drug Reaction Reporting Systems/ OR Abnormalities, Drug-Induced/ OR adverse effects.fs. OR drug effects.fs. OR toxicity.fs. OR chemically induced.fs. OR complications.fs. or (adverse effect* or adverse event* or adverse reaction* or adverse outcome* or drug effect* or drug event* or drug induced or drug reaction* or drug related or chemically induced or complication* or harm* or side effect* or toxicit* or tolerab* or anaemia or anemia or edema or oedema or diarrhea or diarrhoea or abdominal pain* or nausea or vomiting or anorexi* or (loss ADJ2 appetite) or skin rash or sore mouth or sensitive mouth or tiredness or fatigue or muscle pain* or muscle cramp* or neutropenia).ti,ab,kf.

4.2. Embase

exp side effect/ or exp adverse drug reaction/ or exp drug toxicity/ or exp complication/ OR adverse drug reaction.fs. OR complication.fs. OR drug toxicity.fs. OR side effect.fs. or (adverse effect* or adverse event* or adverse reaction* or adverse outcome* or drug effect* or drug event* or drug induced or drug reaction* or drug related or chemically induced or complication* or harm* or side effect* or toxicit* or tolerab* or anaemia or anemia or edema or oedema or diarrhea or diarrhoea or abdominal pain* or nausea or vomiting or anorexi* or (loss ADJ2 appetite) or skin rash or sore mouth or sensitive mouth or tiredness or fatigue or muscle pain* or muscle cramp* or neutropenia).ti,ab,kw.

4.3. PsycINFO

-

4.4. Web of Science

("adverse effect*" or "adverse event*" or "adverse reaction*" or "adverse outcome*" or "drug effect*" or "drug event*" or "drug induced" or "drug reaction*" or "drug related" or "chemically induced" or complication* or harm* or "side effect*" or toxicit* or tolerab* or anaemia or anemia or edema or oedema or diarrhea or diarrhoea or "abdominal pain*" or nausea or vomiting or anorexi* or (loss NEAR/1 appetite) or "skin rash" or "sore mouth" or "sensitive mouth" or tiredness or fatigue or "muscle pain*" or "muscle cramp*" or neutropenia)

4.5. Cochrane

(adverse effect* or adverse event* or adverse reaction* or adverse outcome* or drug effect* or drug event* or drug induced or drug reaction* or drug related or chemically induced or complication* or harm* or side effect* or toxicit* or tolerab* or anaemia or anemia or edema or oedema or diarrhea or diarrhoea or abdominal pain* or nausea or vomiting or anorexi* or loss appetite or skin rash or sore mouth or sensitive mouth or tiredness or fatigue or muscle pain* or muscle cramp* or neutropenia):ti,ab,kw

Supplementary File S2. Quality Assessment

Table S1. Detailed assessment of the methodological quality of the included studies.

Qualitative Studies	1.1	1.2	1.3	1.4	1.5	Total
FIauske, 2019, Norway [23]	Yes	Yes	Yes	Yes	Yes	5.0
Macdonald, 2012, International/USA [36]	Yes	Yes	Yes	Yes	No	4.0
Quantitative randomized controlled trials	**2.1**	**2.2**	**2.3**	**2.4**	**2.5**	**Total**
Adenis, 2014, France [31]	Yes	Yes	Yes	No	No	3.0
Blanke, 2008, International/France [72]	Yes	Yes	Yes	No	Yes	4.0
Blay, 2007, France [28]	No	Yes	No	No	Yes	2.0
Blay, 2015, International/France [73]	Yes	Yes	Yes	No	Yes	4.0
Blay, 2020, International /France [33]	Yes	Yes	Yes	Yes	Yes	5.0
Casali, 2015, International/Italy [63]	Yes	Yes	Yes	No	No	3.0
Dematteo, 2009, International/USA [64]	Yes	Yes	Yes	Yes	No	4.0
Demetri, 2002, International/USA [79]	Yes	No	Yes	No	Yes	3.0
Demetri, 2006, International/USA [117]	Yes	Yes	Yes	Yes	Yes	5.0
Demetri, 2012, International/USA [118]	No	Yes	Yes	Yes	Yes	4.0
Demetri, 2013, International/USA [13]	Yes	Yes	Yes	Yes	Yes	5.0
George, 2009, International/USA [119]	No	No	Yes	No	Yes	2.0
Joensuu, 2012, International/Finland [18]	Yes	Yes	Yes	No	Yes	4.0
Kang, 2013, Japan [74]	Yes	Yes	Yes	Yes	Yes	5.0
Komatsu, 2015, Japan [137]	Yes	Yes	Yes	Yes	Yes	5.0
McAuliffe, 2009, USA [62]	Yes	No	Yes	No	No	2.0
Poole, 2015, International/UK [32]	Yes	Yes	Yes	Yes	Yes	5.0
Reichardt, 2012, International/Germany [75]	No	Yes	Yes	Yes	Yes	4.0
Verweij, 2004, International/The Netherlands [76]	Yes	Yes	Yes	No	Yes	4.0
Verweij, 2007, International/The Netherlands [77]	Yes	Yes	Yes	No	Yes	4.0
Xia, 2010, China [89]	No	Yes	Yes	No	Yes	3.0
Yoo, 2016, Korea [30]	No	Yes	Yes	Yes	Yes	4.0
Quantitative non-randomized	**3.1**	**3.2**	**3.3**	**3.4**	**3.5**	**Total**
Ashraf, 2011, India [59]	Yes	Yes	Yes	No	Yes	4.0
Azribi, 2009, UK [110]	Yes	Yes	Yes	No	Yes	4.0
Ben-Ami, 2016, USA [138]	Yes	Yes	Yes	No	Yes	4.0
Borunda, 2016, Mexico [107]	Yes	Yes	Yes	No	Yes	4.0
Bouche, 2018, France [29]	Yes	Yes	No	No	Yes	3.0
Carbajal-Lopez, 2020, Mexico [34]	Yes	Yes	No	No	Yes	3.0
Chamberlain, 2020, UK [145]	Yes	Yes	Yes	No	Yes	4.0
Chen, 2005, China [99]	Yes	Yes	Yes	No	Yes	4.0
Chen, 2014, Taiwan [135]	Yes	Yes	Yes	No	Yes	4.0
Chu, 2007, USA [136]	Yes	Yes	Yes	Yes	Yes	5.0
Custers, 2015, The Netherlands [22]	Yes	Yes	Yes	No	Yes	4.0
Dematteo,2013, USA [65]	Yes	Yes	Yes	Yes	Yes	5.0

Demetri, 2009, USA [168]	Yes	Yes	Yes	No	Yes	4.0
Den Hollander, 2019, International/The Netherlands [130]	Yes	Yes	Yes	Yes	Yes	5.0
Desai, 2006, USA [126]	Yes	Yes	Yes	No	Yes	4.0
Doyon, 2012, Canada [58]	Yes	Yes	Yes	No	Yes	4.0
Eisenberg, 2009, USA [61]	Yes	Yes	Yes	No	Yes	4.0
Farag, 2017, The Netherlands [111]	Yes	Yes	Yes	Yes	Yes	5.0
Fu, 2018, China [100]	Yes	Yes	Yes	No	Yes	4.0
George, 2012, USA [139]	Yes	Yes	Yes	No	Yes	4.0
Hsiao, 2006, Taiwan [102]	Yes	Yes	Yes	No	Yes	4.0
Hsu, 2014, Taiwan [101]	Yes	Yes	Yes	No	Yes	4.0
Hu, 2020, Taiwan [142]	Yes	Yes	Yes	Yes	Yes	5.0
Hung, 2019, Vietnam [103]	Yes	Yes	Yes	No	Yes	4.0
Italiano, 2013, International/France [90]	Yes	Yes	Yes	Yes	Yes	5.0
Ivanyi, 2020, Germany [147]	Yes	Yes	Yes	No	Yes	4.0
Jiang, 2011, China [69]	Yes	Yes	Yes	No	Yes	4.0
Kanda, 2012, Japan [85]	Yes	Yes	Yes	No	Yes	4.0
Kanda, 2013, Japan [66]	Yes	Yes	Yes	No	Yes	4.0
Kang, 2013, Japan [67]	Yes	Yes	Yes	No	Yes	4.0
Kasper, 2006, Germany [97]	Yes	Yes	Yes	No	Yes	4.0
Kefeli, 2013, Turkey [131]	Yes	Yes	Yes	Yes	Yes	5.0
Kim, 2019, Korea [141]	Yes	Yes	Yes	No	Yes	4.0
Kollar, 2014, Switzerland [144]	Yes	Yes	Yes	No	Yes	4.0
Komatsu, 2015, Japan [137]	Yes	Yes	Yes	Yes	Yes	5.0
Kurokawa, 2017, International/Japan [57]	Yes	Yes	Yes	No	Yes	4.0
Ladha, 2008, Pakistan [114]	Yes	Yes	Yes	No	Yes	4.0
Lee, 2009, Korea [132]	Yes	Yes	Yes	No	Yes	4.0
Li, 2012, China [86]	Yes	Yes	Yes	No	Yes	4.0
Li, 2012, China [134]	Yes	Yes	Yes	No	Yes	4.0
Mannavola, 2007, Italy [127]	Yes	Yes	Yes	N/A	Yes	4.0
Matsumoto, 2011, Japan [129]	Yes	Yes	Yes	No	Yes	4.0
Nishida, 2008, Japan [81]	Yes	Yes	Yes	No	Yes	4.0
Ogata, 2014, Japan [95]	Yes	Yes	Yes	Yes	Yes	5.0
Park, 2009, Korea [104]	Yes	Yes	Yes	No	Yes	4.0
Park, 2016, Korea [116]	Yes	Yes	Yes	No	Yes	4.0
Peixoto, 2018, Portugal [112]	Yes	Yes	Yes	No	Yes	4.0
Poort, 2016, The Netherlands [35]	Yes	Yes	Yes	Yes	Yes	5.0
Prenen, 2006, Belgium [83]	Yes	Yes	Yes	No	Yes	4.0
Raut, 2018, USA [26]	Yes	Yes	No	No	Yes	3.0
Reichardt, 2015, International/Germany [121]	Yes	Yes	Yes	No	Yes	4.0
Reichardt, 2019, International/Germany [68]	Yes	Yes	Yes	No	Yes	4.0
Ruka, 2005, Poland [91]	Yes	Yes	Yes	No	Yes	4.0
Rutkowski, 2012, Poland [122]	Yes	Yes	Yes	Yes	Yes	5.0
Rutkowski, 2018, Poland [84]	Yes	Yes	Yes	No	Yes	4.0
Rutkowski, 2020, Poland [70]	Yes	Yes	Yes	No	Yes	4.0
Ryu, 2009, Korea [80]	Yes	Yes	Yes	No	Yes	4.0
Sahu, 2015, India [123]	Yes	Yes	Yes	No	Yes	4.0
Saito, 2013, Japan [98]	Yes	Yes	Yes	No	Yes	4.0
Sawaki, 2014, Japan [94]	Yes	Yes	Yes	No	Yes	4.0
Schindler, 2005, Germany [96]	Yes	Yes	Yes	No	Yes	4.0
Schlemmer, 2011, Germany [82]	Yes	Yes	Yes	No	Yes	4.0

Schvartsman, 2017, USA [146]	Yes	Yes	Yes	No	Yes	4.0
Serrano, 2019, Spain [92]	Yes	Yes	Yes	Yes	Yes	5.0
Shen, 2017, China [124]	Yes	Yes	Yes	No	Yes	4.0
Shirao, 2010, Japan [125]	Yes	Yes	Yes	No	Yes	4.0
Son, 2017, Korea [140]	Yes	Yes	Yes	No	Yes	4.0
Suresh Babu, 2017, India [106]	Yes	Yes	Yes	No	Yes	4.0
Tielen, 2013, The Netherlands [60]	Yes	Yes	Yes	No	Yes	4.0
Usman, 2007, Pakistan [169]	Yes	Yes	Yes	No	Yes	4.0
Wang, 2012, USA [170]	Yes	Yes	Yes	No	Yes	4.0
Wang, 2020, China [27]	Yes	Yes	Yes	Yes	Yes	5.0
Wolter, 2008, Belgium [128]	Yes	Yes	Yes	No	Yes	4.0
Wong, 2008, UK [93]	Yes	Yes	Yes	No	Yes	4.0
Wu, 2018, China [71]	Yes	Yes	Yes	Yes	No	4.0
Xia, 2020, China [109]	Yes	Yes	Yes	No	Yes	4.0
Yeh, 2017, Taiwan [143]	Yes	Yes	Yes	No	Yes	4.0
Yildrim, 2017, Turkey [113]	Yes	Yes	Yes	No	Yes	4.0
Yin, 2016, China [115]	Yes	Yes	Yes	Yes	Yes	5.0
Yoo, 2013, Korea [105]	Yes	Yes	Yes	Yes	Yes	5.0
Yoon, 2012, Korea [133]	Yes	Yes	Yes	Yes	Yes	4.0
Zalcberg, 2005, International/Australia [78]	Yes	Yes	Yes	No	Yes	4.0
Zhang, 2018, China [108]	Yes	Yes	Yes	No	Yes	4.0
Zhu, 2007, China [88]	Yes	Yes	Yes	No	Yes	4.0
Zhu, 2010, China [87]	Yes	Yes	Yes	No	Yes	4.0

References

168. Demetri, G.D.; Heinrich, M.C.; Fletcher, J.A.; Fletcher, C.D.; Van den Abbeele, A.D.; Corless, C.L.; Antonescu, C.R.; George, S.; Morgan, J.A.; Chen, M.H.; et al. Molecular target modulation, imaging, and clinical evaluation of gastrointestinal stromal tumor patients treated with sunitinib malate after imatinib failure. *Clin. Cancer Res.* **2009**, *15*, 5902–5909. https://doi.org/10.1158/1078-0432.ccr-09-0482.
169. Usman, M.; Syed, N.; Kakepoto, G.; Adil, S.; Khurshid, M. Hematological and nonhematological toxicities of imatinib mesylate in patients with chronic myeloid leukemia and gastrointestinal stromal tumor. *Indian J. Pharmacol.* **2007**, *39*, 192–195. https://doi.org/10.4103/0253-7613.36538.
170. Wang, D.; Zhang, Q.; Blanke, C.D.; Demetri, G.D.; Heinrich, M.C.; Watson, J.C.; Hoffman, J.P.; Okuno, S.; Kane, J.M.; von Mehren, M.; et al. Phase II trial of neoadjuvant/adjuvant imatinib mesylate for advanced primary and metastatic/recurrent operable gastrointestinal stromal tumors: Long-term follow-up results of Radiation Therapy Oncology Group 0132. *Ann. Surg. Oncol.* **2012**, *19*, 1074–1080. https://doi.org/10.1245/s10434-011-2190-5.
